# The New York Medical and Surgical Reporter

**Published:** 1845-12

**Authors:** 


### THE MEDICAL EXAMINER.

### PHILADELPHIA, DEC., 1845.

### THE NEW YORK MEDICAL AND SURGICAL REPORTER.

EDITED BY CLARKSON T. COLLINS, M. D., ETC.

This is a new candidate for professional favour, the chief object of which appears to be to report the doings at the Medical Institutions of New York, and especially at the dispensaries attached to the two schools. We have received the three first numbers, consisting in all of fifty-two pages octavo, or seventeen pages each, with a cover, and learn that it is to appear semi-monthly, that size.

From the brevity of the reports it would not seem to be so much the purpose of the Journal to illustrate the principles of pathology and therapeutics, as to chronicle the number and variety of cases, and names of the prescribers and operators at the several institutions.

The editor thinks that "a Medical Journal, established on the principles that ours is,—(the Reporter)—occupying an entire [entirely] new field, to be made up of American practice mostly, must and will succeed;" and "trusts that every American citizen connected with the Medical Profession in any way, or every one taking an interest in it, will feel a *national pride* in sustaining our [his] Journal!" The editor talks largely of the advantages of the "NATIONAL EMPORIUM" [?]; speaks of "the Journals of *our neighbors* on the other side of the Atlantic"! says "there never was, perhaps, so little interest taken in American Medical Journals, as at the present time." "But," he remarks, "we hope such will not be the case much longer." Well, if it be true that "so little interest is taken in American Medical Journals at the present time," we shall rejoice, and we think our "brethren in the trade" will join us, that an Ajax has entered the field. Freely will we accord to him the arms of some former Achilles, if peradventure we may discover the lion's skin!

